# Single Snapshot Imaging of Optical Properties (SSOP) for Perfusion Assessment during Gastric Conduit Creation for Esophagectomy: An Experimental Study on Pigs

**DOI:** 10.3390/cancers13236079

**Published:** 2021-12-02

**Authors:** Lorenzo Cinelli, Eric Felli, Luca Baratelli, Silvère Ségaud, Andrea Baiocchini, Nariaki Okamoto, María Rita Rodríguez-Luna, Ugo Elmore, Riccardo Rosati, Stefano Partelli, Jacques Marescaux, Sylvain Gioux, Michele Diana

**Affiliations:** 1 Department of Gastrointestinal Surgery, San Raffaele Hospital IRCCS, 20132 Milan, Italy; elmore.ugo@hsr.it (U.E.); rosati.riccardo@hsr.it (R.R.); 2 Research Institute against Digestive Cancer (IRCAD), 67000 Strasbourg, France; nariaki.okamoto@ircad.fr (N.O.); rita.rodriguez-luna@ircad.fr (M.R.R.-L.); jacques.marescaux@ircad.fr (J.M.); michele.diana@ircad.fr (M.D.); 3 Department of Visceral Surgery and Medicine, Inselspital, University of Bern, 3010 Bern, Switzerland; eric.felli@dbmr.unibe.ch; 4 Department of BioMedical Research, Visceral Surgery and Medicine, University of Bern, 3010 Bern, Switzerland; 5 ICube Laboratory, University of Strasbourg, 67400 Strasbourg, France; baratelli@unistra.fr (L.B.); ssegaud@unistra.fr (S.S.); Sylvain.Gioux@intusurg.com (S.G.); 6 Department of Surgical Pathology, San Camillo Hospital, 00152 Rome, Italy; abaiocchini@scamilloforlanini.rm.it; 7 Faculty of Medicine and Surgery, Vita-Salute San Raffaele University, 20132 Milan, Italy; partelli.stefano@hsr.it; 8 Pancreas Translational & Clinical Research Center, Pancreatic Surgery Unit, IRCCS San Raffaele Scientific Institute, 20132 Milan, Italy; 9 Department of General, Digestive and Endocrine Surgery, Nouvel Hôpital Civil, University of Strasbourg, 67000 Strasbourg, France

**Keywords:** esophageal resection, Ivor Lewis, esophagectomy, single snapshot, optical properties, SSOP, perfusion assessment, anastomotic leak, gastric conduit, spatial frequency domain imaging

## Abstract

**Simple Summary:**

Anastomotic leak is the most dangerous complication occurring after esophagectomy and its relationship with inadequate visceral perfusion is widely recognized. Currently, the adequate perfusion of the gastric conduit is intraoperatively assessed by surgeons using subjective indicators (e.g., serosal color or pulsatile flow of vessels). During the last decades, several innovative optical techniques based on the interaction of light with tissue have been developed to monitor perfusion in esophagogastric surgery. However, these innovative approaches are characterized by a lack of video rate and reproducibility. They also provide operator-dependent results and lengthen the surgical workflow. Single Snapshot imaging of Optical Properties (SSOP) is an optical technique, which can overcome such limitations, providing quantitative information on the optical properties of biological tissues over a large field of view. It is the first study to demonstrate the accuracy of SSOP in the quantification of serosal StO_2_% in a porcine gastric conduit model.

**Abstract:**

Anastomotic leakage (AL) is a serious complication occurring after esophagectomy. The current knowledge suggests that inadequate intraoperative perfusion in the anastomotic site contributes to an increase in the AL rate. Presently, clinical estimation undertaken by surgeons is not accurate and new technology is necessary to improve the intraoperative assessment of tissue oxygenation. In the present study, we demonstrate the application of a novel optical technology, namely Single Snapshot imaging of Optical Properties (SSOP), used to quantify StO_2_% in an open surgery experimental gastric conduit (GC) model. After the creation of a gastric conduit, local StO_2_% was measured with a preclinical SSOP system for 60 min in the antrum (ROI-A), corpus (ROI-C), and fundus (ROI-F). The removed region (ROI-R) acted as ischemic control. ROI-R had statistically significant lower StO_2_% when compared to all other ROIs at T15, T30, T45, and T60 (*p* < 0.0001). Local capillary lactates (LCLs) and StO_2_% correlation was statistically significant (R = −0.8439, 95% CI −0.9367 to −0.6407, *p* < 0.0001). Finally, SSOP could discriminate resected from perfused regions and ROI-A from ROI-F (the future anastomotic site). In conclusion, SSOP could well be a suitable technology to assess intraoperative perfusion of GC, providing consistent StO_2_% quantification and ROIs discrimination.

## 1. Introduction

On a worldwide scale, esophageal cancer is ranked eighth in terms of occurrence among all cancers, making it the sixth most common cause of cancer-related mortality [1]. Multimodal treatment includes surgery, radiotherapy, and chemotherapy. Surgery remains the best curative modality for patients with resectable esophageal cancer [2], although the 5-year survival rate ranges from 5 to 47% [3].

Two-stage Ivor Lewis esophagectomy was described for the first time in 1946 [4], becoming the most frequently performed surgical treatment for esophageal cancers located in the medial, distal, or esophagogastric junction of the esophagus [5].

Following this technique, esophagogastric continuity is restored through the anastomosis between the gastric conduit (GC) obtained from the tubulization of the stomach and the remaining esophagus. One of the most serious complications of this type of surgery is anastomotic leakage (AL), occurring in up to 53% of all cases [6,7]. It is associated with a mortality rate of up to 35% [8] and with adverse effects on short-term and long-term outcomes [9,10,11], resulting in an increased recurrence of tumors [12]. Although the etiology of AL is multifactorial [13,14], an insufficient perfusion of the anastomotic site is considered to be a significant and independent predicting factor to determine anastomotic failure [15,16].

Currently, the adequate perfusion of GC is clinically assessed by surgeons using indicators such as serosal color or pulsatile flow of vessels. However, these parameters are subjective and inaccurate to rule out a marginally perfused anastomosis [17]. During the last two decades, several intraoperative optical imaging techniques have been developed and tested to monitor the perfusion of the gastric conduit [18], including laser Doppler flowmetry, near-infrared spectroscopy, laser speckle contrast imaging, infrared thermographic imaging, optical coherence tomography, and indocyanine green (ICG) fluorescence imaging. However, those methods lack reproducibility and/or provide operator-dependent results and/or lengthen the surgical workflow [18,19]. New technologies based on optical properties including multispectral and hyperspectral imaging are progressively implemented in image-guided surgery. This increased interest can be partly explained by the enhanced ability of those endogenous imaging methods to quantify tissue optical properties in terms of physiology and morphology, allowing for improved and reproducible information during interventions, which can influence the surgical decision-making process [20].

Our group has recently assessed gastric tubulization via hyperspectral imaging (HSI) in a survival study on hybrid ischemic preconditioning of the stomach [21]. Additionally, the feasibility and potential interest of HSI during gastrointestinal surgeries, including esophageal resections, has been recently evaluated in preliminary clinical studies [21,22,23,24]. HSI is a contrast-free technology which can provide a chemical quantification of tissue compounds, allowing to quantify tissue oxygen saturation (StO_2_%), among others. Although HSI showed encouraging and consistent results, its main downside remains the lack of video rate, which greatly limits its usability as a real-time surgical navigation tool.

Spatial Frequency Domain Imaging (SFDI) is an optical technique that can provide a 3D corrected profile and quantitative information of optical properties (i.e., absorption and reduced scattering coefficients) of biological tissues over a large field of view [25,26]. The potential of SFDI to provide information related to tissue oxygenation has been demonstrated in the past at both preclinical and clinical levels [27,28,29,30,31]. However, the standard embodiment of SFDI requires several frames to be acquired and it does not provide images in real-time. For this reason, Single Snapshot imaging of Optical Properties (SSOP) was developed to enable a video rate imaging capability in the SFDI, by limiting the number of required frames to only one [32,33,34,35]. The natural drawbacks of this single snapshot technique include a loss of image resolution and the presence of edge artifacts. Nevertheless, with the implementation of the latest Convolution Neural Network (CNNs) architectures [35] and the use of state-of-the-art GPGPU computing, it was possible to achieve significant improvements in the overall image quality and profile correction, while maintaining real-time capabilities [35].

In the present study, our aim is to perform a preliminary validation of SSOP camera to intraoperative quantify serosal StO_2_% in an experimental gastric conduit (GC) model.

## 2. Materials and Methods

### 2.1. Experimental Workflow

First of all, laparotomy was performed and the GC was prepared. After tubulization, the resected region of the stomach was maintained near the GC, still connected to the esophagus. Four ROIs were manually selected as follows: ROI-A (antrum), ROI-C (greater curvature/corpus), ROI-F (fundic region/future anastomotic site), and ROI-R (the resected upper part of the stomach). The post-procedural observational phase was held for 60 min, at 15 min time points (T0, T15, T30, T45, and T60). Following this timeline, for each ROI, the SSOP system was used to detect StO_2_% values and LCLs were measured as biological “ground truth”, together with systemic BGA. At the end of the 60 min observation period, histopathological evaluation and scoring were performed in the same four ROIs. Each animal served as its own control.

**Figure 1:**
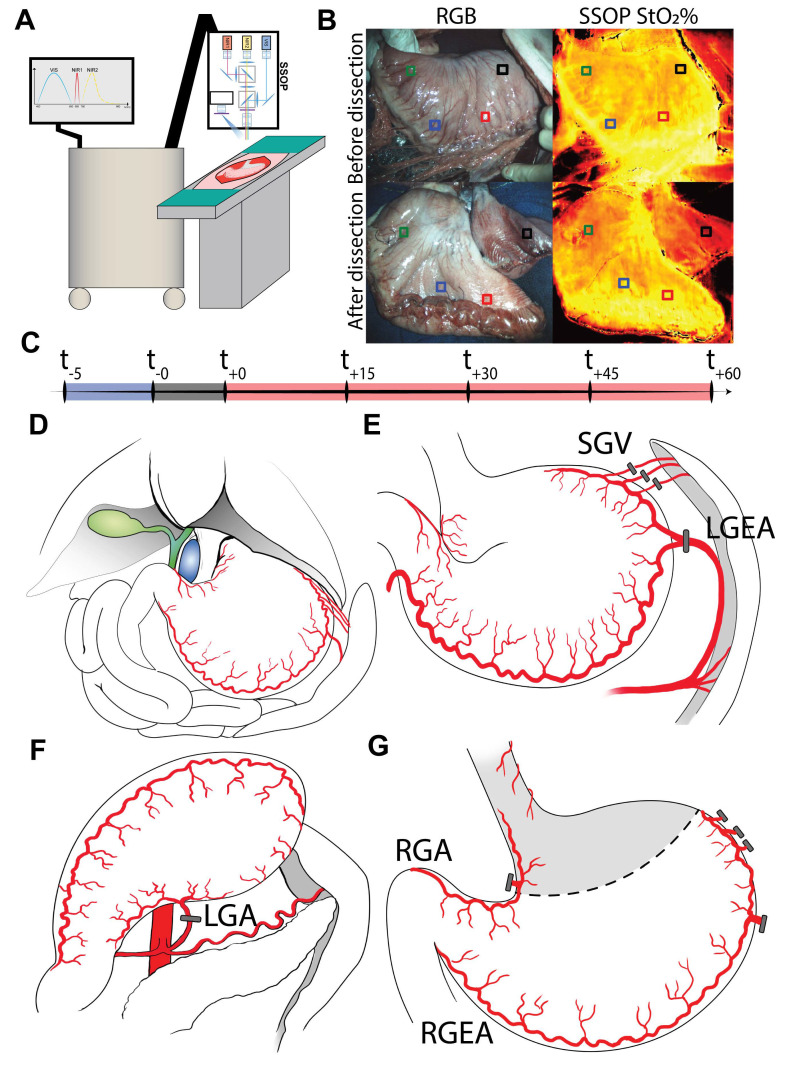
SSOP experimental workflow. (**A**) First of all, the SSOP machine was prepared for data acquisition. (**B**) Regions of interest (ROIs) were marked with surgical stitches before any dissection in order to obtain StO_2_% values and correspondent LCLs in the same ROIs along the entire timeline. Preselection of ROIs for antrum (ROI-A), corpus (ROI-C), fundus (ROI-F), and the future removed region (ROI-R) was essential to prevent any possible selection bias related to postoperative selection. (**C**) The acquisition of data started 5 min before the preparation of the gastric conduit (t_−5_), with the baseline evaluation of the healthy stomach. After gastric conduit (GC) formation, the acquisition proceeded in 15 min time points, for a total of 60 min, which was the clinical estimated time between GC preparation and the packing of the esophagogastric anastomosis. During this period, the upper separated part of the stomach (ROI-R) was supposed to become increasingly ischemic after resection. (**D**) Normal porcine abdominal anatomy. Gastric vascularization is the same in humans, even if the stomach of the pig is two to three times larger and more bag-shaped. The vena cava (blue) and the biliary tree (green) are highlighted. (**E**) Ligation of the left gastroepiploic artery (LGEA) and short gastric vessels (SGV). (**F**) Ligation of the left gastric artery (LGA). (**G**) After transection, the perfusion of the gastric conduit was maintained via the right gastroepiploic arcade (RGEA); the antrum/duodenum was also perfused by the right gastric artery (RGA). ROI-R, even if partially perfused by distal esophageal vessels, received only a minimum amount of blood, enough to consider it ischemic, but not totally devascularized.

The study aimed to predict gastric conduit viability through the analysis of SSOP images in order: (i) to recognize perfused from non-perfused stomach, using ROI-R as ischemic control, (ii) to predict the level of gastric perfusion during the postoperative phase, and (iii) to predict biological data. The hypercube extracted from SSOP images was used to train two CNNs. Finally, the generated artificial intelligence (AI) score for the postoperative phase was generated and the quantitative analysis of SSOP images was correlated with biological and histopathological data (Figure 1A–C).

### 2.2. Sample Size Calculation

Sample size calculation was performed using the correlation between optical and biological data. The calculation derived from previous publications on bowel ischemia, which showed a ρ correlation coefficient of −0.7 [23,24]. The required sample size in terms of paired values was 4, considering α = 0.05 with power (1 − α) = 0.9. In the present study, 120 paired values of StO_2_% and lactates values were obtained in a total of 6 pigs.

### 2.3. Animals

The present study, which was part of the QuantSURG COLORECTAL project (Imagerie optique quantitative pour le guidage du geste chirurgical dans le cancer colorectal), was approved by the local Ethical Committee on Animal Experimentation (ICOMETH 38.2019.01.121) on 3 September 2019, as well as by the French Ministry of Superior Education and Research (MESR) (APAFIS#20819-2019052411591088v3). All animals used in the experiment were managed according to French laws for animal use and care, and according to the directives of the European Community Council (2010/63/EU) and ARRIVE guidelines [36]. Six adult pigs (*Sus scrofa* ssp. *domesticus*, mean weight: 36.5 ± 3.3 kg) were housed and acclimatized for 48 h in an enriched environment, respecting circadian cycles of light–darkness, with constant humidity and temperature conditions. They were fasted 24 h before surgery, with ad libitum access to water, and finally sedated (zolazepam + tiletamine 10 mg/kg IM) 30 min before the procedure in order to decrease stress. Anesthesia was performed intravenously (18-gauge IV catheter in-ear vein) with Propofol 3 mg/kg and maintained with rocuronium 0.8 mg/kg along with inhaled isoflurane 2% via the automatic standard respiratory system. Vital parameters were monitored through a mechanical ventilator machine. Heartbeat was monitored with a pulse oximeter (Mindray PM-60). At the end of the protocol, pigs were euthanized with a lethal dose of Pentobarbital Sodium (40 mg/kg) (Exagon, Axience SAS, Pantin, France).

### 2.4. Surgical Procedure

Through a median laparotomy, the stomach was prepared leaving only the right gastroepiploic and pyloric vessels for its vascular supply. Gastric vessels were ligated using surgical clips (LIGACLIP Multi-Patient-Use Single Clip Appliers, Ethicon, Endosurgery Inc., Cincinnati, OH, USA). Successively, an approximately 4 cm wide GC was created, starting from the greater curvature, and using a surgical stapler (ENDO GIA™ linear stapler equipped with 45 mm black reloads, Medtronic, Minneapolis, MN, USA) (Figure 1D–G).

### 2.5. SSOP Imaging

SFDI is based on the projection of spatially modulated patterns of light on the sample (typically using a Digital Micromirror Device (DMD) system) and on the acquisition with a camera of the diffused backscattered light arising from it. In its simplest configuration, SFDI requires at least six frames: two different spatial frequency patterns (e.g., f_x_ = 0 mm^−1^, f_x_ = 0.2 mm^−1^) and three-phase shifts for each of them. The phase-shifted sequence is then used to demodulate the signal, hence obtaining the modulation amplitude maps of the given sample at each spatial frequency [25]. Using a calibration step involving the measurement of a calibration phantom with known optical properties, it is then possible to compute the diffuse reflectance maps, i.e., R_DC_ (f_x_ = 0 mm^−1^) and R_AC_ (f_x_ = 0.2 mm^−1^). Finally, solving the inverse problem using a light propagation model, here a Monte Carlo-based Look-Up Table (LUT) [37] algorithm, allows to retrieve the optical properties of the sample (i.e., absorption and reduced scattering coefficients) for each pixel of the image. Due to the need for at least six frames, SFDI cannot perform in real-time. Instead, SSOP allows to sensibly reduce acquisition time and to reach video rate performances, thanks to a single high spatial frequency frame required to extract the optical properties of the sample. The main difference in the workflow in comparison with SFDI consists of using a Fourier transform-based filtering technique for the demodulation step [33]. After the extraction of the modulation amplitude maps, the processing workflow is the same as for SFDI. It is worth noticing that degradation in image quality and the presence of edge artifacts are the main consequences of the use of a single frame. However, significant improvements have been achieved in recent years by first optimizing the filtering technique [38] and by adopting Deep Neural Network approaches for the demodulation [35]. Additionally, the latest developments on SSOP also account for a tridimensional profile correction of the sample in order to reduce the quantification error of SFDI associated with a variation of light intensity across non-flat sample surfaces [35].

### 2.6. Deep Learning Method for SSOP

In this study, the latest deep learning optimized SSOP workflow was adopted, as described by Aguénounon et al. [35]. In a nutshell, the standard Fourier domain filtering demodulation was replaced by two dedicated CNNs based on a U-Net architecture as follows: the former was dedicated to the extraction of the modulation amplitude of the signal for each spatial frequency, and the latter was used for the profilometry analysis. Both networks were trained using high quality images obtained with SFDI acquisition sequences (with 7 phase shifts instead of 3 to enhance quantification accuracy and image quality) and optimized for efficient and low-cost computation performances, by reducing the number of parameters and layers involved at a minimum, and subsequently allowing for a real-time high visual quality optical property quantification for up to 1 MP images. The training dataset consisted of a total of 200 high quality images divided into n = 40 images of tissue-mimicking silicone phantoms with different optical properties ranging from μ_a_ = 0.005 to 0.05 mm^−1^ for absorption and from μ’_s_ = 0.5 to 3 mm^−1^ for reduced scattering; *n* = 52 images of hands from different Caucasian men and women in various configurations; *n* = 108 images from ex vivo and in vivo swine organs in several orientations (stomach, small bowel, colon, kidney, pancreas, liver, and spleen).

### 2.7. Instrumentation

**Figure 2:**
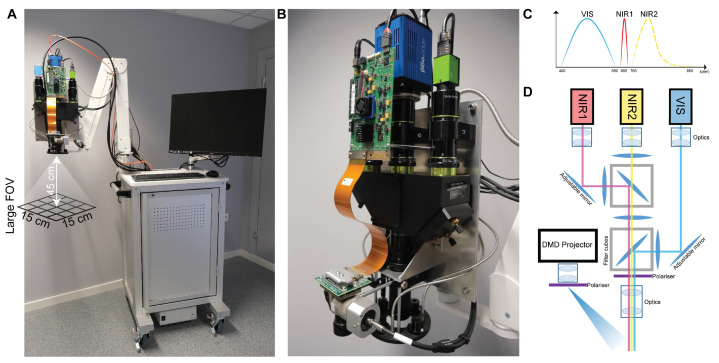
SSOP descriptive figure. (**A**) Preclinical cart for fluorescence, SFDI, and SSOP imaging in the operating room. The imaging head is mounted onto an articulated arm for an easy positioning over the surgical table at the desired working distance. The main body of the cart contains the laser sources, the white light lamp, and the PC workstation. (**B**) Imaging head based on a trident architecture to enclose three separate channels for RGB, NIR1, and NIR2 imaging. A DMD-based projection system is also mounted onto the head for the generation of structured illumination over the FOV. The light source is delivered to the projector via a fiber-based coupling system. An illumination ring is used to homogeneously deliver white light illumination of the surgical field and fluorescence excitation wavelength. (**C**) Schematics of the imaging bands for the trident, namely: the RGB camera (JAI GO-5000C-USB, JAI Ltd., Kanagawa, Japan) covers the VIS bandwidth (400–650 nm); the NIR1 camera (pco.pixelfly USB, Excelitas PCO GmbH, Kelheim, Germany) covers the first near-infrared bandwidth (centered around 665 nm); and the NIR2 camera (pco.edge 5.5, PCO AG, Excelitas PCO GmbH, Kelheim, Germany) covers the second near-infrared bandwidth (700–900 nm). (**D**) Schematics of the optical path in the trident for the co-registration of the three imaging channels, together with the configuration of the DMD-based projector. A pair of filtering cubes are used to isolate the NIR1 and NIR2 channels, and a set of mirrors are used to align the FOV of the three cameras.

The imaging system (Figure 2A,B) included a white light plasma lamp (Thorlabs Inc., Newton, NJ, USA) for the illumination of the surgical field, together with a fiber-coupled custom-made high-power two wavelengths laser source working at 665 and 860 nm. The projection of sinusoidal patterns on the sample was performed with a Digital Micromirror Device-based projector (Vialux GmbH, Chemnitz, Sachsen, Germany). The field-of-view (FOV) of the system was slightly greater than 15 × 15 cm at a working distance of 45 cm as shown in Figure 2A. The imaging head was based on a 3-channel architecture: 2 near-infrared monochrome cameras (PCO.edge 5.5 and PCO.pixelfly USB, Excelitas PCO GmbH, Kelheim, Germany) were used for the acquisition of the SSOP frames with 1024 × 1280 pixel resolution at 665 and 860 nm, and an RGB camera (JAI GO-5000C-USB, JAI Ltd., Kanagawa, Japan) was also added to record the surgical field (Figure 2C,D). The co-registration of the scene by the cameras was obtained thanks to a customized optomechanical system in which optical filters were included to separate the 3 channels at the collection side. In addition, a pair of linear polarizers (PPL05C, Moxtek, Orem, UT, USA) were used in crossed configurations at the projection and camera sides to reject the specular reflections originating from the sample surface. The working distance of the imaging system was 45 ± 5 cm, hence offering a comfortable operating condition for the physicians during acquisition. Oxygenation computation was achieved applying Lambert–Beer’s law for chromophore absorption inside the biological tissue [39].

### 2.8. Blood Analysis

LCLs were obtained through a full-thickness puncture of the gastric wall, in correspondence with the preselected ROIs. A blood drop was obtained and a portable strip-based lactate analyzer (EDGE, Apex Bio, Taipei, Taiwan) was used to quantify LCL levels, with a margin error of 0.35 mmol/L. The correlation analysis of data was performed between StO_2_% parameters detected via the SSOP system and LCLs concentration. Systemic blood was sampled through a central catheter placed in the right external jugular vein (6 French IV catheter) and evaluated through a handheld wireless blood tester (Epoc^®^ Blood Analysis System, Siemens Healthcare GmbH, Erlangen, Germany). These systemic BGA were used to monitor the surgical intervention to rule out any bias in the post-procedural observation of the GC. BGA measured pO_2_, pCO_2_, pH, base excess, bicarbonate, hemoglobin, hematocrit, glucose, creatinine, urea, BUN, electrolyte, and lactates.

### 2.9. Histology

A first gastric full-thickness biopsy was taken before the surgical procedure as healthy control. This control biopsy (CB) was removed from the posterior side of the future removed region of the stomach while the other four biopsies were collected in the selected ROIs 60 min after the GC formation. Sections of 5 μm were taken from formalin-fixed paraffin-embedded blocks and were dewaxed and rehydrated before staining at room temperature. A treatment with a hematoxylin Harris formula (Leica Biosystems, Nussloch GmbH, Heidelberg, Germany) for 10 min and then a wash with acid alcohol for 2 s and tap water for 2 min were performed. Eosin staining with Eosin 0.5% (Leica Biosystems) for 3 min was performed before washing with tap water for 30 s. Finally, the sections were dehydrated with ethanol 100% and placed in xylene until their mounting with coverslips. A semi-quantitative blinded analysis was performed by an expert pathologist using the following score and variables: necrosis, neutrophils infiltration, and congestion, with a score from 0 to 3 (none, mild, moderate, and severe, respectively).

### 2.10. Statistical Analysis

Statistics were performed using GraphPad 8.3 (GraphPad Software, San Diego, CA, USA). A Pearson’s rho was analyzed to perform the correlation between optical and biological data. All data were expressed as means ± standard error (SEM). One-way and two-way ANOVA with Dunnett’s multiple comparisons were performed for parametric tests to calculate differences in continuous paired variables. A two-tailed *p*-value < 0.05 was considered statistically significant. Principal component analysis (PCA) was used to cluster the population of data from 4 ROIs. StO_2_% and LCL variables were used for the analysis. The PCA method was based on pre-standardization of the data with the largest eigenvalues. Finally, data were labeled as from the experimental design (ROI-A, ROI-C, ROI-F, and ROI-R).

## 3. Results

### 3.1. Blood Gas Analysis

The systemic blood gas analysis (BGA) supported the consistency of the experimental workflow and the healthy condition of pigs, showing only minimal changes over time when compared to T0. The full dataset and comparison are available in Table S1.

### 3.2. Histologic Analysis

**Figure 3:**
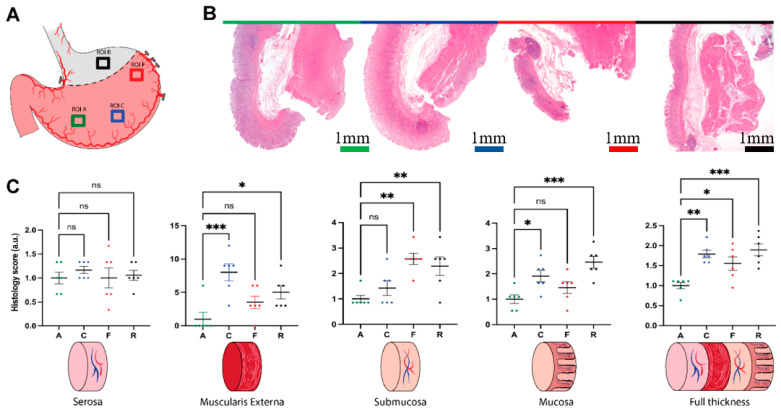
Histopathological assessment. (**A**) ROIs localization map. (**B**) Hematoxylin and eosin staining of biopsies of all ROIs 60 min after tubulization, green (antrum, ROI-A), blue (corpus, ROI-C), red (fundus–future anastomotic site, ROI-F), black (resected region, ROI-R). Magnification 0.125×. Scale bar 1 mm. Pictures were sampled using microscope Zeiss AXIO scope A1. (**C**) Statistical analysis of the histology score, per single layer, and full-thickness. Data were expressed as mean and ±SEM and normalized with the antrum dataset. One-way ANOVA was used, ns *p*  >  0.05, * *p*  ≤  0.05, ** *p*  ≤  0.01, *** *p*  ≤  0.001 *N* = 6.

Histological analysis of the biopsies of the selected regions of interest (ROIs) showed a progressive alteration of the cell morphology of the gastric wall layers. This aspect was more evident in the mucosa, characterized by superficial erosions and strong congestion, edema, and inflammatory components such as neutrophils and eosinophils, especially in ROI-R (removed region). Muscularis mucosae presented light and homogeneous alterations due to inflammation. Edema and congestion were present in the submucosae, while in ROI-R the muscularis externa presented a higher inflammatory infiltration which involved the serosa too. Nevertheless, the serosa showed no important alteration between ROIs (Figure 3A,B). Control biopsies are available in Figure S1. Overall, histological alterations have grown gradually from ROI-A (antrum), ROI-C (corpus), and ROI-F (fundus) up to ROI-R. Statistical analysis was reported in Table S2. After 60 min of ischemia, the serosa showed non-statistically significant alterations in all ROIs (Figure 3C). The muscularis externa presented a significantly higher damage in ROI-C and ROI-R when compared to ROI-A (*p* = 0.0004, *p* = 0.0373, respectively). The submucosa showed a statistically significantly higher histological alteration, mostly due to inflammation in ROI-F and ROI-R (*p* = 0.0013, *p* = 0.0073, respectively), while the mucosa showed an increased and significant damage in ROI-C and ROI-R (*p* = 0.0211, *p* = 0.0004, respectively). The average of the full-thickness biopsy damage presented a statistically significant increase going from ROI-A to ROI-C (*p* = 0.0010), ROI-F (*p* = 0.0187), and ROI-R (*p* = 0.0003), accordingly with the histopathological observation.

Additional analysis on neutrophils infiltration is reported in Figure S2.

### 3.3. Local Capillary Lactates Quantification

**Figure 4:**
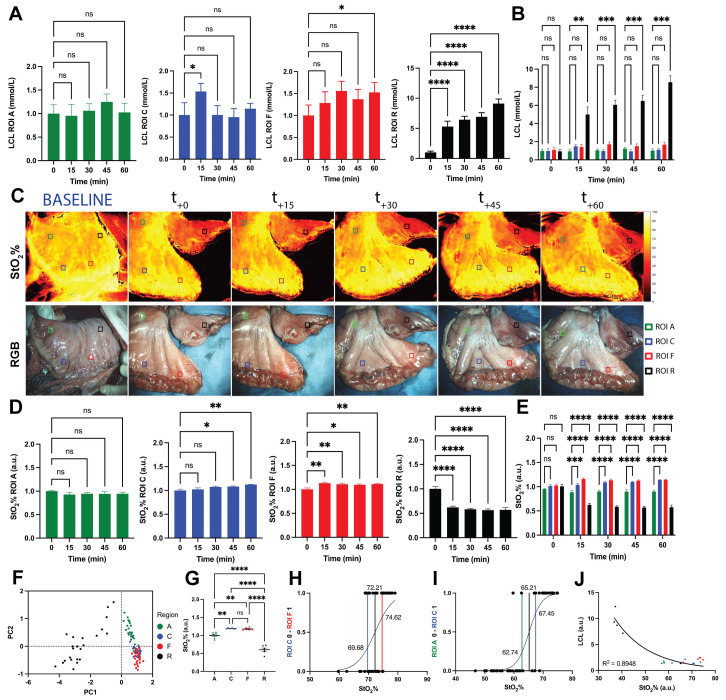
LCLs and StO_2_% quantification and analysis. Different colors indicate different ROIs: green (ROI-A), blue (ROI C), fundus (ROI-F), and black (ROI-R). (**A**) Evolution of LCLs for single ROIs. Only the removed region showed statistically significantly higher values of LCLs for each time point from T15 to T60 when compared to T0; the corpus showed this relationship for T0 vs. T15 and the fundus for T0 vs. T60. Data were normalized for T0 of ROI-A. (**B**) LCL comparison between ROIs. After gastric conduit formation, the removed region showed statistically significantly higher values of LCLs when compared to the other gastric regions. Data were normalized for T0 of ROI-A. (**C**) SSOP images showing StO_2_% of the gastric conduit and the removed region from baseline to T60, and correspondent true color (RGB) images. (**D**) Oxygenation trends for each region of interest during the full ischemic period with a non-constant sampling rate. Between T0 (when the GC is complete) and T15, the sampling rate is around 10 s to monitor the dynamics of tissue perfusion, whereas for t = −5 min (baseline) and T30, T45, and T60, a single point was sampled. Data were normalized for T0 of ROI-A. (**E**) Evolution of StO_2_% for single ROIs. StO_2_% increased along time in the corpus (T45 and T60) and fundus (T15, T30, T45, and T60). Instead, as expected, StO_2_% values at T15, T30, T45, and T60 were lower than T0. Data were normalized for T0 of ROI-A. (**F**) Principal component analysis of the ROIs. The variables selected are LCL and StO_2_% and the label used is the ROI (A, C, F, R). ROI-R represents a separate cluster from the other ROIs. Principal Component (PC)1 and PC2 contribute to 94.83% of the variance. (**G**) StO_2_% comparison between ROIs. After GC formation, the removed region showed lower StO_2_% values than other gastric districts. Instead, StO_2_% of the antrum appeared lower when compared to the corpus and fundus. Data were normalized for T0 of ROI-A. (**H**) Simple logistic regression analysis between ROI-A and ROI-F. (**I**) Simple logistic regression analysis between ROI-A and ROI-C. (**J**) Pearson’s correlations between LCL and StO_2_%. Higher LCL measurements correlated with lower StO_2_% values. Data were extracted at T60 and normalized for the respective ROI-A. All data are expressed as mean and ± SEM. For every statistical analysis, a two-tailed *p*-value < 0.05 was considered significant. One-way ANOVA was used, ns *p* >  0.05, * *p*  ≤  0.05, ** *p*  ≤  0.01, *** *p*  ≤  0.001, **** *p*  ≤  0.0001. *N* = 6.

Mean local capillary lactate (LCL) values were not significantly different between the four ROIs at T0, upon the formation of the GC, as expected. ROI-A had similar LCLs for each time point, while ROI-C and ROI-F showed significant differences in terms of T0 vs. T15 (1.00 ± 0.28 mmol/L vs. 1.54 ± 0.18 mmol/L, *p* = 0.0307) and T0 vs. T60 (1.00 ± 0.24 mmol/L vs. 1.53 ± 0.22 mmol/L, *p* = 0.0451) respectively. Regarding ROI-R, LCL levels obtained at T15, T30, T45, and T60 were statistically significantly higher than T0 (*p* < 0.0001) (Figure 4A).

The comparison performed between different ROIs showed that mean lactate levels collected at ROI-A were significantly lower than those sampled at ROI-R at T15 (0.95 ± 0.24 mmol/L vs. 5.31 ± 0.89 mmol/L, *p* = 0.0091), T30 (1.06 ± 0.15 mmol/L vs. 6.44 ± 0.56 mmol/L, *p* = 0.0003), T45 (1.25 ± 0.17 mmol/L vs. 6.91 ± 0.69 mmol/L, *p* = 0.0007), and T60 (1.02 ± 0.20 mmol/L vs. 9.11 ± 0.76 mmol/L, *p* = 0.0002) (Figure 4B). The same statistical relationship was shown by ROI-C and ROI-F when compared to ROI-R. Instead, lactate levels were not significantly different between ROI-A vs. ROI-C, ROI-A vs. ROI-F, and ROI-C vs. ROI-F (Figure 4B). LCLs were standardized for ROI-A lactate values at T0. Statistical analysis is reported in Table S3.

### 3.4. SSOP-Based StO_2_% Quantification

SSOP images of StO_2_% are shown in Figure 4C. ROI-A had no differences in terms of StO_2_% for each time point. Instead, ROI-C showed statistically significant lower T0 values than T45 (1.00 ± 0.03 vs. 1.08 ± 0.01, *p* = 0.0334) and T60 (1.00 ± 0.03 vs. 1.13 ± 0.01, *p* = 0.0010). Regarding ROI-F, StO_2_% at T0 remained significantly lower when compared to T15, T30, T45, and T60 values (*p* = 0.0012, *p* = 0.0080, *p* = 0.0181, *p* = 0.0049, respectively). Regarding ROI-R, StO_2_% was statistically higher at T0 when compared to T15, T30, T45, and T60 (*p* < 0.0001) (Figure 4D). StO_2_% curves of all pigs are available in Figure S3.

The comparison between different ROIs showed no differences for T0. ROI-R had statistically significant lower StO_2_% values when compared to all other ROIs at T15, T30, T45, and T60 (*p* < 0.0001). However, ROI-C and ROI-F showed higher values than ROI-A at T15 (ROI-C vs. ROI-A, *p* = 0.0007; ROI-F vs. ROI-A, *p* < 0.0001), T30 (*p* < 0.0001), T45 (*p* < 0.0001), and T60 (*p* < 0.0001) (Figure 4E, Table S4).

### 3.5. Correlation between StO_2_% Values and LCLs

LCL values were normalized for LCL measurements of ROI-A at T0. Principal component analysis (PCA) of StO_2_% and LCL values after resection showed ROI-R as a separated cluster from the other ROIs. ROI-A and ROI-F appeared as separate groups while ROI-C was overlapped between ROI-A and ROI-F (Figure 4F). Similarly, data were normalized for ROI-A at T60 to investigate the power of the camera in discriminating between ROIs. As a result, measurements evaluated at ROI-A showed statistically significant differences when compared to ROI-C (*p* = 0.0013), ROI-F (*p* = 0.0028), and ROI-R (*p* < 0.0001). A significant difference was found for ROI-C vs. ROI-R (*p* < 0.0001) and ROI-F vs. ROI-R (*p* < 0.0001) while no difference was found regarding ROI-C vs. ROI-F (Figure 4G).

A simple logistic regression analysis was used to find the cut-off value to discriminate ROI-A from ROI-C and ROI-C from ROI-F. The threshold value to discriminate between ROI-C and ROI-F was 72.21%, with an AUC of 0.7274 (95% CI 0.5847 to 0.8702, *p* = 0.0069) (Figure 4H) while the threshold value to distinguish between ROI-A and ROI-C was found to be 65.21%, with an AUC of 0.9688 (95% CI 0.9275 to 1.0000, *p* < 0.0001) (Figure 4I). StO_2_% and LCLs were found to be negatively correlated (R = −0.8439, 95% CI −0.9367 to −0.6407, *p* < 0.0001) (Figure 4J).

## 4. Discussion

In the current experimental study, the SSOP imaging system was used to evaluate StO_2_% values corresponding to selected ROIs, using the resected upper part of the stomach (ROI-R) as ischemic control.

ROI-R showed lower StO_2_% values when compared to other ROIs. Interestingly, the corpus and fundus maintained StO_2_% values higher than the antrum, probably due to the inflammatory process activated during the manipulation of the stomach. Gastric manipulation, the sequential ligation of vessels and the division of the stomach, are factors which can affect the physiology of healthy gastric tissue. Akiyama et al. [40] already described that the formation of the gastric conduit induces severe changes in microcirculation, particularly in the anastomotic region of the gastric fundus.

As a result of this manipulation, immune cells promote the acute inflammatory response by increasing the permeability of vessels and allowing the secretion of various cytokines and chemokines. Neutrophils are the first ones to be involved, by secreting vasoactive and pro-inflammatory mediators, including histamine, platelet-activating factors (PAFs), bradykinin, and thrombin. These mediators increase vascular permeability, promoting fluid accumulation (edema) and leukocyte extravasation (Figure S2). Polymorphonuclear neutrophils produce reactive oxygen species (ROS), responsible for endothelial dysfunction by oxidation of crucial cellular signaling proteins such as tyrosine phosphatases [41]. The presence of ROS together with the increased permeability of vessels could account for higher StO_2_% values detected by the present experimental model, reflecting the growing trend of inflammation along time and from the antrum to the fundus. As described above, inflammation is part of the regular process of healing. However, persistent inflammation led to excessive quantities of pro-inflammatory macrophages, whereas the number of macrophages with anti-inflammatory phenotypes became lower. As a result, the establishment of a highly inflammatory environment with an overabundance of inflammatory mediators promotes the degradation of the extracellular matrix, preventing anastomotic healing [42].

The StO_2_% values provided by means of SSOP negatively correlated with LCL values, confirming the biological validity of the method. Instead, no correlation was found between StO_2_% and the histopathologic analysis. The limited period of ischemia (60 min), together with the residual perfusion of ROI-R from esophageal vessels, were probably accountable for non-significant histological changes in the gastric tissue. Nevertheless, this time frame is consistent since it is similar to the time between GC formation and the packing of the esophagogastric anastomosis in the Ivor Lewis procedure performed in humans.

Our group has previously used HSI and a system called HYPER (HYperspectral-based Enhanced Reality) to assess StO_2_% and intraoperatively localize preselected ROIs during esophagectomy [43], small bowel ischemia [24], and hepatectomy [44]. HYPER allowed to compare HSI to LCLs and mucosal scan with confocal laser endomicroscopy (CLE) on the same ROI [43] with high accuracy. However, the time currently necessary for HSI acquisition is around 10 s and still not yet fully “real-time”.

Instead, fluorescence angiography with indocyanine green (ICG) allows for the real-time visualization of tissue vascularization, showing promising results for the evaluation of perfusion in numerous surgical procedures, hence leading to modifications in the surgical strategy and consequently to a decrease in the rates of AL [45,46]. However, the ICG interpretation is subjective while the quantitative evaluation of ICG is a widely unexplored field. In addition, serious adverse events after intravenous application of ICG are described, such as anaphylactic shock, up to sudden deaths in very rarely reported cases [47].

Several other methods for perfusion assessment are reported in the literature. However, they lack in terms of quantitative parameters, provide arbitrary perfusion units, and are influenced with vascular heterogeneity [18].

As a result, non-invasiveness, the absence of adverse events, quantification properties, and the video rate acquisition of the SSOP technology are advantages worthy of being highlighted. The real-time imaging capabilities of SSOP have already been demonstrated [39]. However, the configuration used for the present experiment did not allow to reach minimum requirements required for high frame rate acquisitions. It was due to the high integration times required on the imaging side (>100 ms). Further development of the laser sources has already been started to achieve better performance on the illumination side, hence allowing shorter exposure times during acquisitions in the next experimental steps.

Additionally, the potential SSOP system’s ability to detect and quantify inflammation could make this new technology a useful tool to screen suffering tissues that are more likely to develop delayed anastomotic healing, moving beyond the concept of perfusion alone.

The main limitations of this study lie in the animal model, the very limited sample size, and the acute nature of the experiment. In addition, the absence of gold standard values of tissue oxygenation and inflammation makes it difficult to compare SSOP with other methods. Finally, SSOP has not yet been adapted to endoscopic systems, resulting in a lack of potential usefulness for an increasingly widespread minimally invasive surgery (MIS). Actually, a prototype based on a laparoscopic surgical instrument is being developed to bring SSOP to MIS, and it is soon going to be ready for validation via pre-clinical trials on other swine models.

## 5. Conclusions

The present study has demonstrated for the first time the safety and reliability of SSOP technology in determining the real-time adequate perfusion of GC when compared to a certain ischemic gastric tissue, providing a quantitative evaluation through StO_2_% values, without exogenous fluorophores. SSOP is a suitable intraoperative tool which could potentially predict AL in the clinical setting. This preliminary validation provided a robust dataset useful to compare single snapshot images with the video rate as the next experimental step. Further studies are needed to validate the system and to develop a minimally invasive setup, to finally proceed to clinical application.

## Data Availability

Data presented in this study are available in the article and Supplementary Materials.

## Supplementary Materials

The following data are available online at https://www.mdpi.com/article/10.3390/cancers13236079/s1, Figure S1: Control biopsy of the stomach (T0), Figure S2: Neutrophils distribution (A) and quantification (B), Figure S3: StO_2_% curves of all pigs, Table S1: Blood gas analysis quantification, Table S2: Histological assessment and correlation, Table S3: LCL quantification and correlation, Table S4: StO_2_% quantification and correlation.
