# Supplementary material for: Single Snapshot Imaging of Optical Properties (SSOP) for Perfusion Assessment during Gastric Conduit Creation for Esophagectomy: An Experimental Study on Pigs
Source: Cancers (Basel). 2021 Dec 2;13(23):6079. doi: 10.3390/cancers13236079 (PMC8656795; doi:10.3390/cancers13236079)
Supplement: Supplementary file 1 [file cancers-13-06079-s001.zip › cancers-1476082 supplementary.pdf]

cancers

MDPI

Single Snapshot Imaging of Optical Properties (SSOP) for Perfusion Assessment during Gastric Conduit Creation for Esophagectomy: An Experimental Study on Pigs

Lorenzo Cinelli, Eric Felli, Luca Baratelli, Silvère Ségaud, Andrea Baiocchini, Nariaki Okamoto, María Rita Rodríguez-Luna, Ugo Elmore, Riccardo Rosati, Stefano Partelli, Jacques Marescaux, Sylvain Gioux and Michele Diana

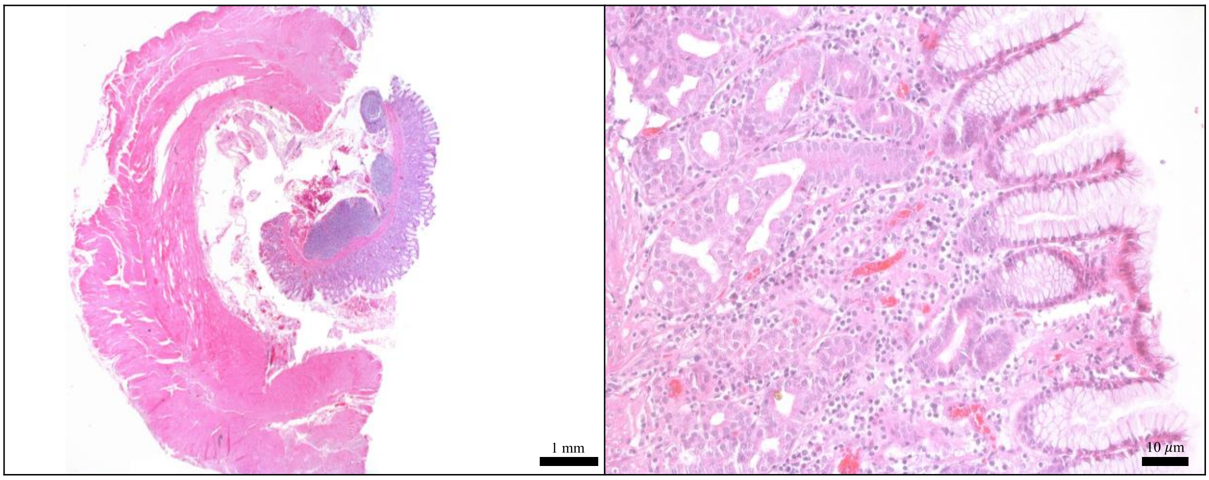

Figure S1. Control biopsy of the stomach (T0). Hematoxylin and Eosin staining of ROI-R (Pig 1) before the tubulization (T0). Both full thickness (left) and mucosae (right) magnification show no alteration of the tissue before surgery.

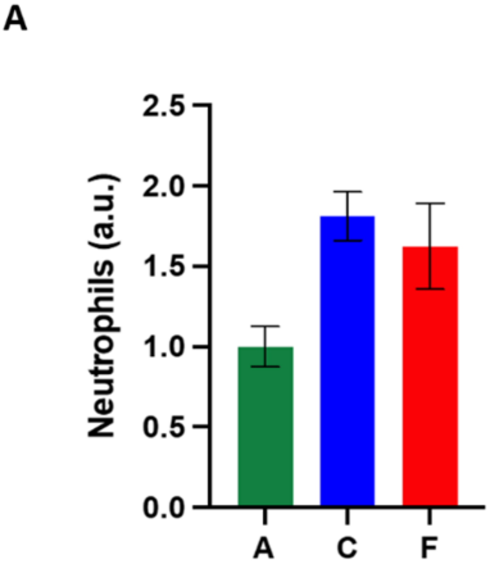

B

	A	C	F
Number of values	6	6	6
Minimum	0.3750	1.500	0.7500
25% Percentile	0.9375	1.500	1.031
Median	1.125	1.688	1.688
75% Percentile	1.125	2.250	2.063
Maximum	1.125	2.250	2.625
Mean	1.000	1.813	1.625
Std. Deviation	0.3062	0.3687	0.6567
Std. Error of Mean	0.1250	0.1505	0.2681
Lower 95% CI	0.6787	1.426	0.9358
Upper 95% CI	1.321	2.199	2.314

A = antrum (ROI-A)
C = corpus (ROI-C)
F = fundus (ROI-F)

Figure S2. Neutrophils distribution (A) and quantification (B). The overall neutrophils number in corpus and fundus was 81.3% and 62.5% higher than antrum, respectively. Data are expressed as mean and ± SEM and normalized for ROI-A.

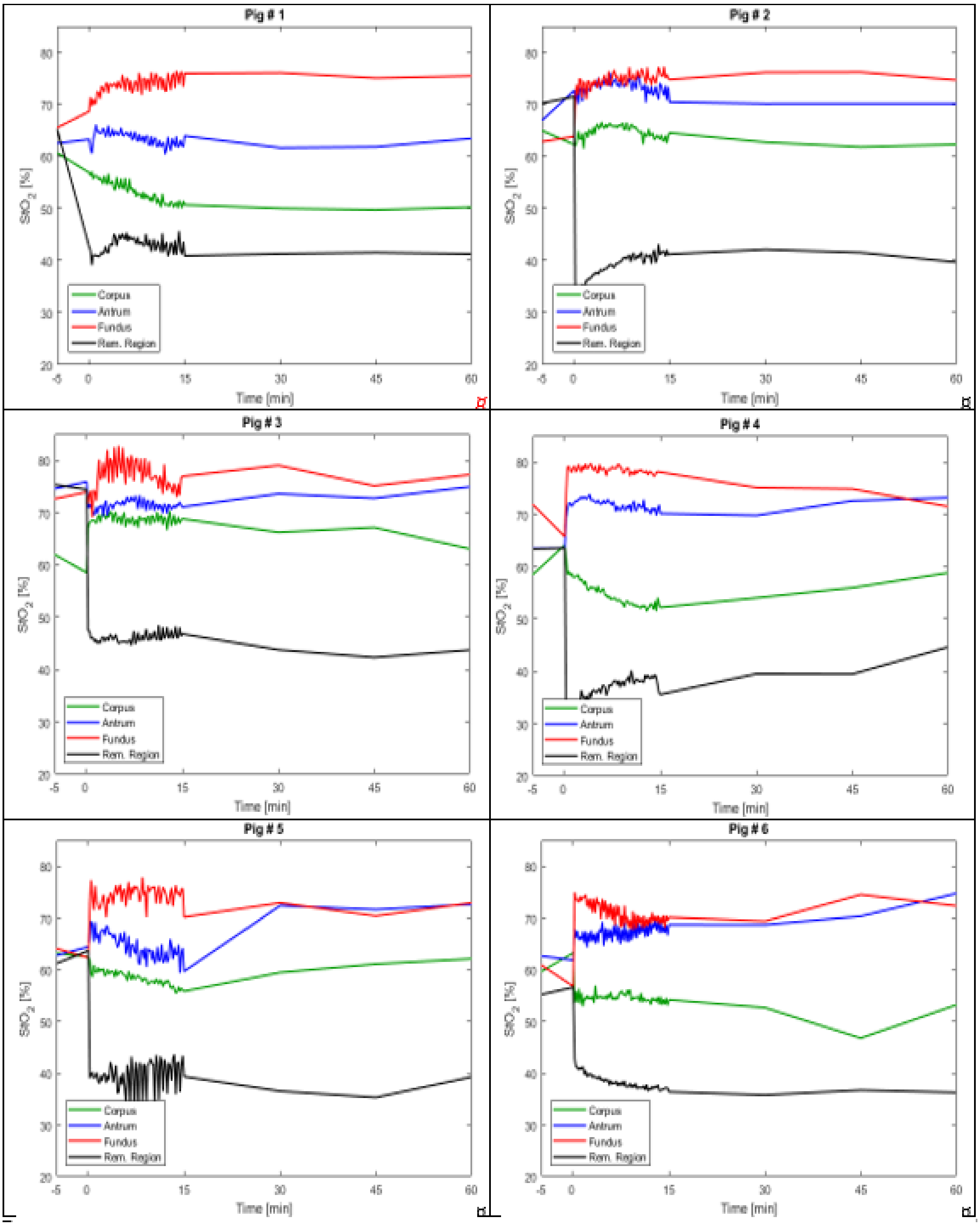

Figure S3. StO2% curves of all pigs. StO2% measurements at ROI-R (black) are lower than ROI-A (green), ROI-C (corpus) and ROI-F (fundus) at T15, T30, T45, and T60. Instead, after GC completion, StO2% values increase from corpus to fundus.
